# One-step nanopatterning of conjugated polymers by electron-beam-assisted electropolymerization

**DOI:** 10.1093/jmicro/dfv013

**Published:** 2015-03-29

**Authors:** Takeshi Higuchi, Hidetoshi Nishiyama, Mitsuo Suga, Hirohmi Watanabe, Atsushi Takahara, Hiroshi Jinnai

**Affiliations:** 1 Takahara Soft Interfaces Project, Exploratory Research forAdvanced Technology (ERATO), Japan Science and Technology Agency (JST), 744 Motooka, Nishi-ku, Fukuoka 819-0395, Japan; 2 Institute for Materials Chemistry and Engineering (IMCE), Kyushu University, 744 Motooka, Nishi-ku, Fukuoka 819-0395, Japan; 3 JEOL Ltd., 1-2 Musashino 3-chome, Akishima, Tokyo 196-8558, Japan

**Keywords:** nanopatterning, conjugated polymer, electropolymerization, atmospheric scanning electron microscopy

## Abstract

We propose a one-step nanopatterning method where liquid monomers are polymerized directly with an electron beam under an atmospheric pressure. The method allows precise positional control of an electron beam that induces electropolymerization based on an anodic oxidation only in the irradiated areas. Various versatile conjugated polymers, including polypyrrole, polyaniline and poly(3-hexylthiophene), have been directly polymerized from monomers without solvents and patterned by our one-step nanopatterning method. Vertically oriented arrays of nanorods several hundred nanometers in diameter with an aspect ratio (height to diameter) of around two were fabricated.

## Introduction

Since the discovery of the conductive polymer polyacetylene by Shirakawa *et al.* [1,2], conjugated polymers have received much attention because of their tremendous potential for organic electronic devices, such as organic transistors [3,4], organic light-emitting diodes [5] and organic photovoltaics [6–8]. Various kinds of conjugated polymers and their derivatives have been studied, including polyacetylene, polyfluorene, polypyrrole, polyaniline, polythiophene and poly(*p*-phenylene vinylene). Meanwhile, nanotechnology has stimulated the development of a wide variety of nanofabrication techniques for creating fine nanoarchitectures, including dots, lines and perpendicularly oriented nanorods [9,10]. In particular, nanorod arrays are promising nanostructures for the various applications such as sensors [11], catalysis [12], data storage [13], in addition to the above-mentioned purposed, i.e., light-emitting diodes [14], and photovoltaic devices [15]. These arrays have been fabricated by filling perpendicular pores with a target material [16–18] or by nanoimprinting the materials [19,20].

The pore filling method requires the preparation of a template with perpendicular pore arrays that is then filled with the material. Subsequently, the template is removed to leave the rod array. Although the resulting nanorod arrays may be highly ordered with flexible size control, the pore filling method is a complicated multi-step (minimum two-step) protocol. Moreover, a new template is required each time to fabricate a different pattern. The pore filling method is applicable to produce the nanopatterns at industrial scale owing to parallel processing; however, it requires good formability of the target materials, e.g. the conjugated polymers, to fill the template. Therefore, when they have no solubility to solvents and exhibit high glass transition temperature, the pore-filling techniques may be difficult to use in the nanopatterning.

Because most conjugated polymers actually have low solubility to solvents commonly used in nanofabrication (e.g. chloroform, toluene, etc.), they need to be chemically modified to increase the solubility. Although the nanopatterns of substituted conjugated polymers have been assembled by the pore-filling [21] or nanoimprinting [22] methods, nanopatterning of unsubstituted conjugated polymers such as polypyrrole is difficult by using these template methods due to their low solubility and high glass transition temperature. Therefore, it would be ideal if one could create nanopatterns of various conjugated polymers without chemical modification from their monomers, i.e. without solvents, at a desired position on a substrate.

Besides the pore-filing methods, energetic particles, such as electrons and ions, have been widely used for generating nanostructures in electron-sensitive resist films [23–25]. Electron-beam and focused ion-beam lithography are representative methods of direct nanopatterning and tend to be used to produce two-dimensional master patterns for photolithography. Because the resist film must be placed in a specimen chamber under high vacuum in this method, it is difficult to use liquid monomers of conjugated polymers as precursors in conventional electron-beam lithography. Recently, Ahn *et al.* [26] used X-rays and electron beams to solidify 3-hexylthiophene (3HT), which was condensed on a cryogenically cooled substrate, to generate a film of 3HT oligomer on a micrometer scale. They assumed that the reaction mechanism was similar to that of conventional electropolymerization. The growth reaction was terminated at fewer than six monomers because of the low mobility of the monomers at the cryogenic temperature (the monomer was solidified at the low temperature), and also because the electropolymerization was conducted in a high vacuum environment to preserve the intensity of the X-ray and electron beam. Nevertheless, electron beam irradiation may be useful to polymerize neat conjugated monomers directly. Therefore, an instrument, enabling us to irradiate electron beam to the neat monomers at liquid state, would be a promising tool to synthesize conjugated polymers because the growth reaction is easily occurred at liquid state as compared with that at solid state.

Atmospheric scanning electron microscopy (ASEM) has recently become available to the scientific community [27]. It allows samples to be observed under atmospheric conditions, and thus allows liquid specimens, such as electrolytes, colloidal suspensions and cells, to be analyzed [27,28]. Electrons are passed through a 100-nm-thick silicon nitride (SiN) membrane, and the reflected electrons from the samples are detected for imaging. Although ASEM is designed to observe specimens, the instrument can also be used as a tool for irradiating liquid monomers with electrons to induce polymerization without solvents (hereafter referred to as ‘electron-beam-assisted electropolymerization’).

In this study, we simultaneously polymerized liquid conjugated monomers without solvents and nanopatterned the polymerized conjugated polymers by electron beam irradiation (one-step nanopatterning) using ASEM. The monomers such as pyrrole, 3HT and aniline were used to demonstrate the novel concept. Vertically oriented nanorods on a substrate (nanorod arrays) were fabricated from various conjugated polymers, including polypyrrole, P3HT and polyaniline.

## Methods

### Electron-beam-assisted polymerization of conjugated monomers

The liquid conjugated monomer [pyrrole, aniline or 3HT (TCI)] was degassed by freeze–pump–thaw cycles, and a drop (20 μl) was placed on a specimen dish containing a SiN membrane (thickness: 100 nm) for ASEM (JASM-6200, JEOL). The monomers were irradiated with an electron beam (acceleration voltage: 10–30 kV; beam current: 35–515 pA) from the bottom of the droplets through the SiN membrane. The irradiated region was controlled by the scanning unit installed in the ASEM. After electron beam irradiation, the excess monomer was washed away with acetonitrile and ethanol.

### Nanopatterning of conjugated polymers

Nanorods were prepared from the liquid monomer (pyrrole, aniline or 3HT) by injecting a spot electron beam for 0.5–2 s. The excess monomer was washed away with acetonitrile and ethanol. The nanorods were coated with Os and then observed by field emission-scanning electron microscopy (FE-SEM, S-5200, Hitachi; acceleration voltage: 5 and 20 kV).

### Characterization of P3HT by FT-IR absorption microspectroscopy

The P3HT fabricated on a SiN membrane by irradiation with electron beam (referred to as EB-P3HT) was characterized by FT-IR absorption microspectroscopy (VERTEX 70 and HYPERION 2000, Bruker) performed at beamline BL43IR of SPring-8 (Japan) at room temperature in air. A fine-focused IR beam from the synchrotron radiation was narrowed to 10 × 10 µm through an aperture. The IR spectrum of the P3HT sample was acquired in transmission mode.

### Electrical properties of P3HT measured by atomic force microscopy

The electrical properties of EB-P3HT and a chemically synthesized regioregular P3HT reference were measured with current sensing-atomic force microscopy (CS-AFM, 5500AFM; Agilent Technologies). The P3HT sample (10 × 15 × 2 µm) was prepared on a glass substrate coated with a vapor deposited Al film. Au-coated cantilevers (OMCL-TR400PB-1, Olympus Corporation; spring constant: 0.09 N m^−1^) were used to measure the current between the tip and the Al film. The EB-P3HT was doped with iodine by immersion in an aqueous solution containing 0.1 M KI and I_2_ for 1 h.

## Results and discussion

**Fig. 1.**
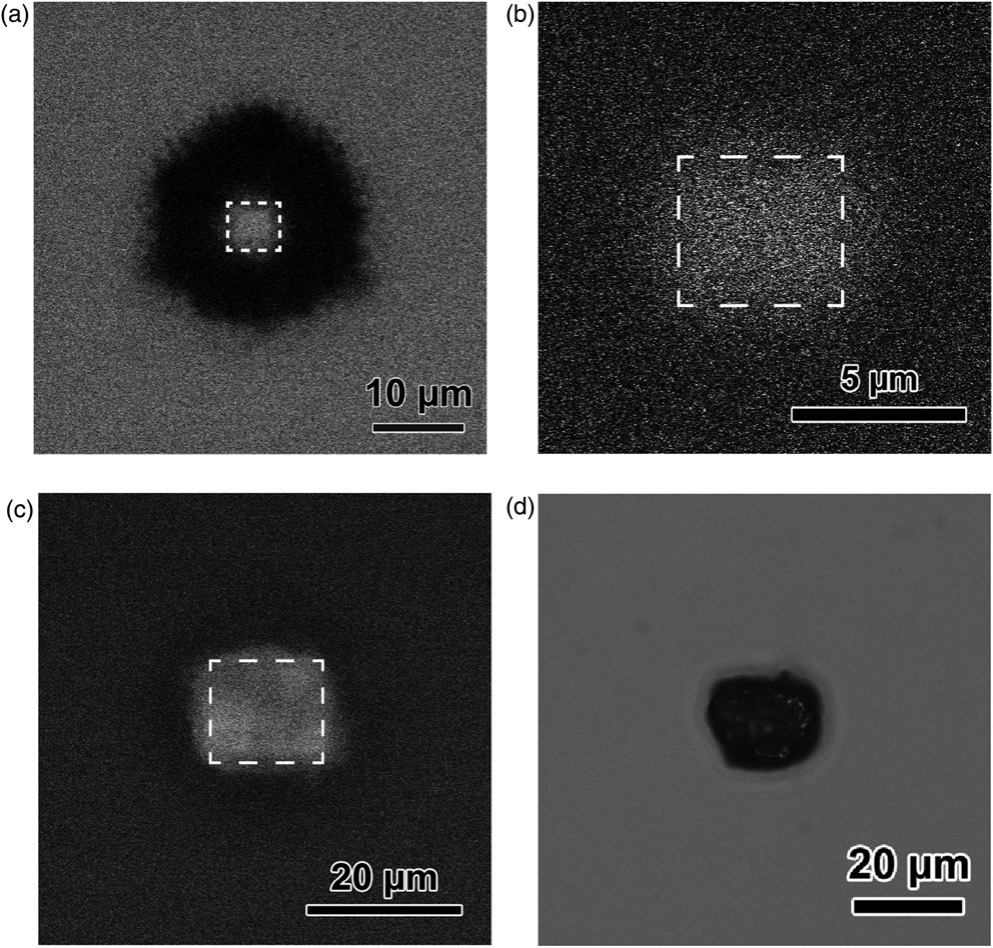
Reaction of conjugated monomers by electron beam irradiation. ASEM images of (a) pyrrole, (b) aniline and (c) 3HT after electron beam irradiation. Dashed lines indicate the regions scanned with the electron beam. (d) Optical microscope image of the structure formed by 3HT by electron beam irradiation.

The pyrrole liquid monomer, which is readily polymerized by electropolymerization, was irradiated with a 30 kV electron beam at room temperature under an atmospheric pressure. Electron beams with a current density of 0.6–10 mA cm^−2^ were used to induce the polymerization of monomers (see Supplementary data online, Fig. S1) [29]. The electron beam was scanned over a region of 1.3 × 1.0 µm. After irradiation, an ASEM image was acquired using a less intense electron beam with a current density of <1 µA cm^−2^ (Fig. 1a). The central part of Fig. 1a appeared brighter than the outer region. During the irradiation, the intensity of the reflected electrons gradually increased over several minutes. Because the intensity of reflected electrons of the ASEM image depends on the electron density of materials, a material with a higher electron density than pyrrole was formed in the irradiated region (indicated by the dashed square in Fig. 1a). The electron beam irradiation was also applied to liquid aniline and 3HT monomers. Figure 1b and c show the ASEM images of aniline and 3HT after electron beam irradiation, respectively. Similar to the pyrrole, the regions irradiated with the electron beam showed a higher electron density in the ASEM images. After the residual 3HT monomer was removed, a film-like substance with dimensions of 18 × 14 µm was observed with an optical microscope (Fig. 1d). The materials produced from three different monomers were not soluble and remained on the SiN substrate after rinsing several times with their good solvents, such as chloroform and dichloromethane for P3HT. The remaining substances were conjugated polymers that showed crosslinking (the spectroscopy results will be discussed later). It is remarkable that the neat liquid monomers reacted with the electron beam; an electrolyte solution of the monomer is used in conventional electropolymerization.

These results suggest that the reaction of the liquid monomer was caused by the electron beam irradiation at a high current density and that controlled irradiation would allow us to perform micro and nanopatterning. Recently, Kuwabata *et al.* [30] have demonstrated the direct nanopatterning of three-dimensional (3D) structures from a non-volatile ionic liquid containing a polymerizable group by using a focused ion beam. Although this method is similar to our method in terms of the irradiation of a liquid precursor with energetic particles, the method reported by Kuwabata *et al.* is more or less limited to precursors with no vapor pressure because they must be placed in a high vacuum chamber.

**Fig. 2.**
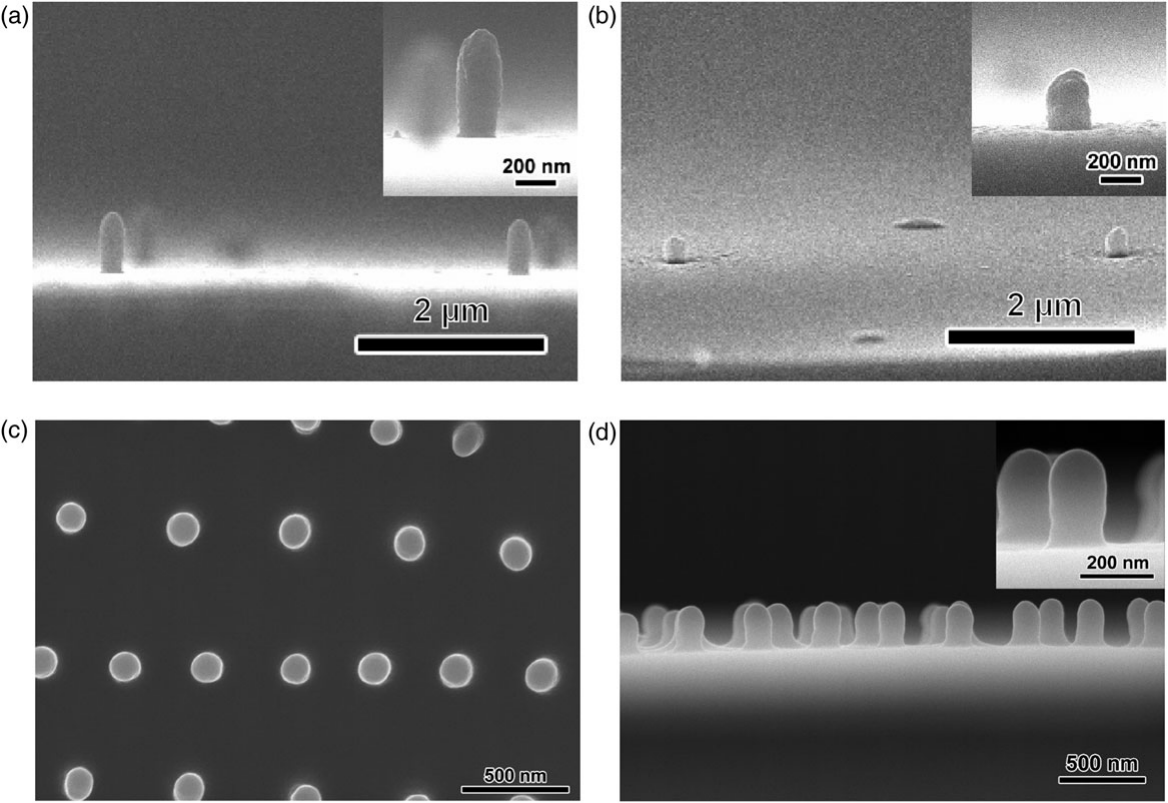
FE-SEM images of conjugated polymer nanorods fabricated by a spot beam. The side views of the (a) polypyrrole and (b) polyaniline nanorods. (c) Top and (d) side view of the EB-P3HT nanorods. The insets are high magnification images of the nanorods.

To explore the potential of our electron-beam-assisted polymerization for nanopatterning, the spot electron beam was injected into the monomers for a few seconds instead of scanning. The FE-SEM images of the structures formed from pyrrole on the SiN membrane after the removal of unreacted monomer are shown in Fig. 2a. In the irradiated regions, nanorods were formed perpendicular to the SiN membrane. At each irradiated spot, the beam was applied for 0.5 s and each spot was separated by ∼9 µm. The diameter and height of the nanorods were ∼250 and 680 nm, respectively. The maximum aspect ratio of the nanorods was 2.7. The side view (inset in Fig. 2a) showed that the nanorods had round tips, implying that the electron density profile in the liquid pyrrole was significant during the polymerization. Because the injected electrons were scattered inside the liquid, the electron density decreased as the depth from the SiN membrane and the distance from the center of the electron beam increased. The size of nanorods could be controlled by changing the irradiation conditions of the electron beam (see Supplementary data online, Fig. S2 for details). The diameter of the nanorods depended on the spot size of the electron beam, and the height varied with the electron beam acceleration voltage, because the penetration depth of the electron beam from the surface of the SiN membrane depends on the acceleration voltage. In addition, the nanorods were fabricated from the liquid monomers on Al- or ITO-coated SiN membranes as long as the electron beam penetrates these membranes to reach the monomers.

The nanopatterning with the spot electron beam was applied to aniline and 3HT. As shown in Fig. 2b–d, the nanorods were successfully prepared from these monomers by irradiations of the spot electron beam with similar electron densities and irradiation times of the pyrrole case. The aspect ratios of nanorods from aniline and 3HT were 1.5 and 2.1, respectively. The aspect ratios of nanorods depended on the monomer, which may be because of the reactivity of monomers with the electron beam. The oxidation potential of pyrrole is lower than that of 3HT, indicating that the pyrrole is polymerized more easily than 3HT [31,32]. In the case of aniline, an insulating polyaniline is formed by electropolymerization in a neutral solution, which implies that the reactivity of aniline is the lowest of the three monomers. Thus, the reactivity of monomers is in the order pyrrole > 3HT > aniline. In the electron-beam-assisted electropolymerization, the nanorods from pyrrole have the highest aspect ratio because of its high reactivity, whereas aniline formed the nanorods with the smallest aspect ratio. Note that the nanorods were formed from aniline even though polyaniline synthesized in a neutral solution by conventional electropolymerization is an insulator. In the conventional electropolymerization, the synthesized polyaniline is deposited on the electrode as the polymerization reaction proceeds. The insulating polyaniline deposited on the electrode inhibits further polymerization of aniline. Therefore, polyaniline has to be electropolymerized in low pH condition (∼1.0) to form the conductive polyaniline by doping of protons. On the other hand, in the case of electron-beam-assisted electropolymerization, the nanorods formation is achieved in neat aniline within seconds. Because the nanorods formation does not require continuous polymerization, electron-beam-assisted electropolymerization may allow fabricating the nanorod even from insulating polyaniline.

**Scheme 1.**
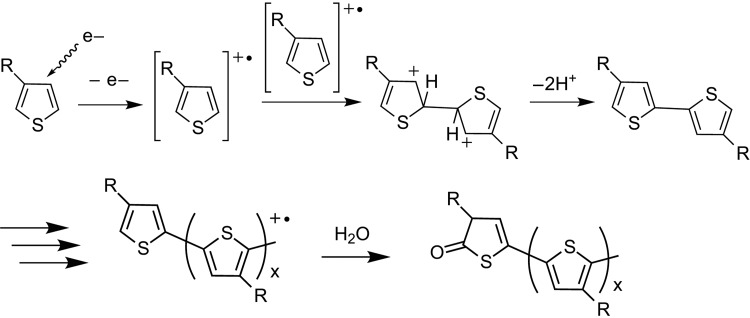
Reaction mechanism of P3HT synthesis by electron beam irradiation. The reaction mechanism is based on that of electropolymerization.

Although the reaction mechanism of the conjugated monomers under electron beam irradiation has not been fully clarified, it is thought that the reaction mechanism of the electron-beam-assisted electropolymerization is similar to that of conventional electropolymerization based on anodic oxidation [26,33]. To investigate the versatility and formation mechanism of our polymerization method, an electropolymerizable monomer used for cathodic reduction, poly(*p*-phenylene vinylene), was also examined. Because *p*-xylylene-α,α′-bis(triphenylphosphonium chloride), the precursor of poly(*p*-phenylene vinylene) [34], is solid at room temperature, we dissolved it in toluene when we irradiated and scanned the electron beam. Even at the highest current density available to the ASEM instrument (i.e. 41.9 mA cm^−2^), no insoluble polymer was formed. These results imply that the formation mechanism of the insoluble polymers was electropolymerization based on an anodic oxidation. A possible reaction scheme is shown in Scheme 1.

**Fig. 3.**
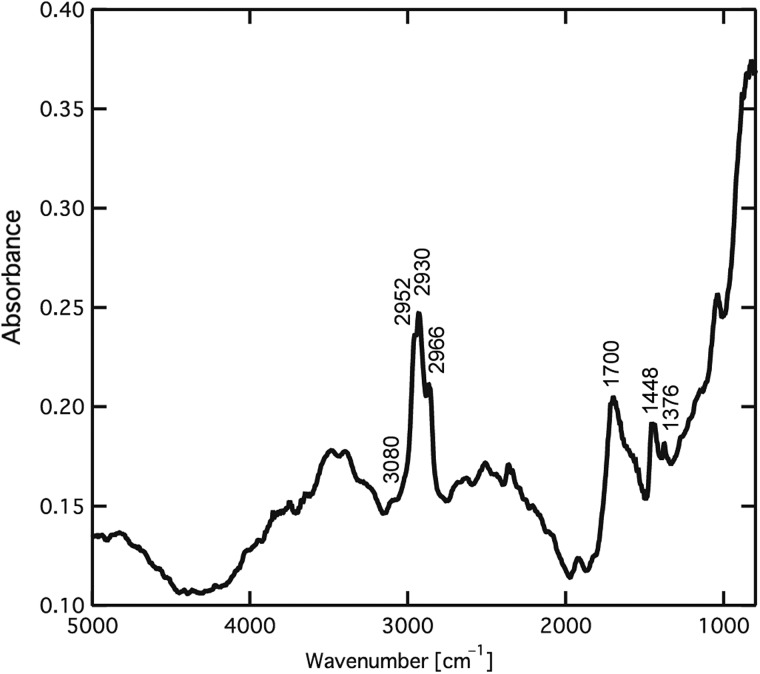
FT-IR spectrum of EB-P3HT films prepared by electron beam irradiation at 0.6 mA cm^−2^.

To investigate the polymerization mechanism further, the polymers were characterized. P3HT was characterized because it is an important conjugated polymer for device fabrication because of its environmental stability, field-effect mobility and good solubility for common organic solvent [35]. P3HT is often also used as the donor layer in organic photovoltaic devices [36–39]. The insoluble film on SiN after electron beam irradiation (Fig. 1c) was characterized with FT-IR microspectroscopy. Figure 3 shows the film's FT-IR spectrum, which was acquired in transmission mode. Hotta *et al.* [40] performed spectroscopic studies of aliphatic-substituted polythiophenes, and they reported that the absorption peaks of chemically synthesized P3HT occurred at 3054 cm^−1^ (C–H stretching vibration of the thiophene ring), 2954, 2923 and 2853 cm^−1^ (C–H bonds on the aliphatic side chain), 1509 and 1456 cm^−1^ (stretching vibrations of the thiophene ring), 1376 cm^−1^ (CH_3_ deformation vibration) and at 824 cm^−1^ (C–H out-of-plane vibration of the 2,3,5-trisubstituted thiophene ring). In the FT-IR spectra of the film on the SiN membrane, the absorption peak appeared at 3080 cm^−1^, corresponding to the C–H stretching vibration of the thiophene ring. The peaks at 2952, 2930 and 2966 cm^−1^ were from the C–H bonds on the aliphatic side chain. The stretching vibration of the thiophene ring and the CH_3_ deformation vibration appeared at 1448 and 1376 cm^−1^, respectively. The C–H out-of-plane vibration of the 2,3,5-trisubstituted ring at 824 cm^−1^ in Fig. 3 was missing due to the large increase in the baseline. These results suggest that the film formed by the electron beam irradiation was composed of oligomers or polymers of 3HT. The peak at 1700 cm^−1^ arose from the C=O stretching vibration, suggesting that the thiophene ring was partially oxidized due to the termination reaction with H_2_O, as described in Scheme 1. The broad peak ∼3400 cm^−1^ and shoulder peak ∼1600 cm^−1^ corresponded to the O–H stretching and bending vibrations, respectively, which was derived from water included in the 3HT and water physically adsorbed on the remaining film because the FT-IR measurement was carried out under ambient conditions. The electron beam initiates the oxidation of the thiophene ring to a cation radical through the loss of a π-electron from the thiophene ring. Two cation radicals couple, and two protons are eliminated to neutralize the charge on the thiophene rings. The polymer chain grows through these reactions. Finally, the cation radical is terminated with H_2_O, which oxidizes the end thiophene ring. The carbonyl group in the FT-IR spectrum is consistent with the oxidation termination reaction.

In addition to the FT-IR measurement, the film formed by the electron beam irradiation was also characterized with visible absorption and photoluminescence microspectroscopies. The visible absorption and photoluminescence spectra of the films are shown in Supplementary data online, Fig. S3. The absorption peak appears ∼470 nm and photoluminescence peak appears ∼600 nm when the film was excited by the light ranging from 450 to 490 nm, indicating that the film is composed of conjugated compound. As compared with the spectra of oligomers [26] and polymers of 3HT [29], the peak position of the film is similar to those of polymers. Taking the results of FT-IR, visible absorption and photoluminescence spectroscopies into considerations, the film formed by the electron beam irradiation was P3HT.

Whitten *et al.* [33] detected protons while condensed benzene was irradiated with an electron beam on a cryogenically cooled substrate, which is consistent with the electropolymerization mechanism based on anodic oxidation. Recently, we have reported cathode luminescence from several liquid aromatic compounds, such as benzene, benzyl ether and anisole, during electron beam irradiation in the ASEM system [41]. The cathode luminescence from the aromatic compounds shows increase in the conjugation length by the electron beam, similar to 3HT. Therefore, electron-beam-assisted electropolymerization could be used for any compound containing π-electrons to generate polymers.

Mass spectrometry was used to determine the molecular weight of EB-P3HT. When the micrometer-sized EB-P3HT film was immersed in chloroform, the film maintained its shape. The lack of solubility suggested that the P3HT was partially cross-linked during the electron-beam-assisted polymerization. Although almost insoluble, the tiny amount of dissolved P3HT was sufficient to obtain a spectrum, which shows that the film contained P3HT molecules that were longer than 10 3HT units (see Supplementary data online, Fig. S4). Furthermore, the electrical properties of EB-P3HT were evaluated by CS-AFM, the detailed results of which are described in Supplementary data online, Fig. S5.

## Concluding remarks

In summary, we have demonstrated a new concept of the direct one-step nanopatterning of various conjugated polymers from liquid monomers without solvents by electron beam irradiation. The reaction mechanism is similar to electropolymerization based on an anodic oxidation, electron-beam-assisted electropolymerization. Vertically aligned nanorods from polypyrrole, P3HT and polyaniline were fabricated by controlled irradiation of liquid monomers with a spot electron beam. Despite the polymerization of aniline is difficult in neutral condition, the electron-beam-assisted electropolymerization makes it possible to fabricate the nanorods from aniline. This result implies that the electron-beam-assisted electropolymerization technique may overcome the limitation of pH condition. The diameter and height of nanorods depended on the spot size and the acceleration voltage of the electron beam, respectively. Reducing the size and increasing the height of the nanorods require a smaller electron beam at a higher acceleration voltage, which can be achieved by using better electron optics; for example, a FE electron gun [42] instead of a tungsten filament gun. Despite some limitations related to instruments, such as the fine collimation of the electron beam, and those related to materials, such as controlling stereo regularity in P3HT, electron-beam-assisted electropolymerization could potentially be a flexible, fast, 3D nanopatterning technique. Although a fabrication technique based on an electron beam irradiation is inadequate to produce with high throughput, one-step nanopatterning would be a valuable new tool for nanoscience and nanotechnology. Precisely fabricated nanorod arrays of conjugated polymers could be used in various organic electronic devices, such as sensors and field-effect transistors. In particular, P3HT nanorods may have great potential for organic photovoltaic devices and would assist in the development of alternative energy resources.

## Supplementary data

Supplementary data are available at http://jmicro.oxfordjournals.org/ online.

## Funding

H.J. gratefully acknowledges financial support received through Grant-in-Aid no. 24310092 from MEXT, Japan. Funding to pay the Open Access publication charges for this article was provided by Japan Science and Technology Agency.
